# Effectiveness of three commonly used transition phase diets in the inpatient management of children with severe acute malnutrition: a pilot randomized controlled trial in Malawi

**DOI:** 10.1186/s12887-017-0860-6

**Published:** 2017-04-26

**Authors:** Christian J. Versloot, Wieger Voskuijl, Sara J. van Vliet, Meta van den Heuvel, Jane C. Carter, Ajib Phiri, Marko Kerac, Geert Tom Heikens, Patrick F. van Rheenen, Robert H. J. Bandsma

**Affiliations:** 1 Department of Pediatric Gastroenterology, Hepatology and Nutrition, University of Groningen, University Medical Center Groningen, Hanzeplein 1, 9700 RB Groningen, The Netherlands; 2 Department of Pediatrics and Child Health, College of Medicine, Blantyre, Malawi; 3 Department of Population Health, London School of Hygiene and Tropical Medicine, London, UK; 4 Leonard Cheshire Disability & Inclusive Development Centre, Department of Epidemiology & Public Health, University College London, London, UK

**Keywords:** Carbohydrate malabsorption, Diarrhea, F75, F100, Ready-to-use therapeutic food, SAM

## Abstract

**Background:**

The case fatality rate of severely malnourished children during inpatient treatment is high and mortality is often associated with diarrhea. As intestinal carbohydrate absorption is impaired in severe acute malnutrition (SAM), differences in dietary formulations during nutritional rehabilitation could lead to the development of osmotic diarrhea and subsequently hypovolemia and death. We compared three dietary strategies commonly used during the transition of severely malnourished children to higher caloric feeds, i.e., F100 milk (F100), Ready-to-Use Therapeutic Food (RUTF) and RUTF supplemented with F75 milk (RUTF + F75).

**Methods:**

In this open-label pilot randomized controlled trial, 74 Malawian children with SAM aged 6–60 months, were assigned to either F100, RUTF or RUTF + F75. Our primary endpoint was the presence of low fecal pH (pH ≤ 5.5) measured in stool collected 3 days after the transition phase diets were introduced. Secondary outcomes were duration of hospital stay, diarrhea and other clinical outcomes. Chi-square test, two-way analysis of variance and logistic regression were conducted and, when appropriate, age, sex and initial weight for height Z-scores were included as covariates.

**Results:**

The proportion of children with acidic stool (pH ≤5.5) did not significantly differ between groups before discharge with 30, 33 and 23% for F100, RUTF and RUTF + F75, respectively. Mean duration of stay after transitioning was 7.0 days (SD 3.4) with no differences between the three feeding strategies. Diarrhea was present upon admission in 33% of patients and was significantly higher (48%) during the transition phase (*p* < 0.05). There was no significant difference in mortality (*n* = 6) between diets during the transition phase nor were there any differences in other secondary outcomes.

**Conclusions:**

This pilot trial does not demonstrate that a particular transition phase diet is significantly better or worse since biochemical and clinical outcomes in children with SAM did not differ. However, larger and more tightly controlled efficacy studies are needed to confirm these findings.

**Trial registration:**

ISRCTN13916953 Registered: 14 January 2013.

## Background

Severe Acute Malnutrition (SAM) affects an estimated 20 million children worldwide and results in high mortality rates of up to half a million child deaths under the age of five annually [1, 2]. SAM is defined by a weight-for-height Z-score greater than three below the mean (based on the 2006 WHO growth standards), a mid-upper arm circumference (MUAC) of less than 115 mm or by the presence of nutritional edema [3]. Severe acute malnutrition differs from severe chronic malnutrition, which manifests as stunting [1]. Severely malnourished children without complications are currently treated as outpatients in community-based management (CMAM) programmes using specially formulated ready-to-use therapeutic food (RUTF) [3]. However, children with complicated SAM (i.e. SAM plus the presence of ‘danger signs’ such as fever, absent appetite, pneumonia, dehydration, severe edema) require inpatient treatment. Therapeutic feeding for these children involves three phases. In the stabilization phase*,* life-threatening problems are treated, the child is metabolically stabilized and a low-protein milk-based formula diet, ‘F-75’, is provided. Once appetite is regained, feeds are being finished and edema has started to resolve, the child will be gradually transitioned to a diet with a higher protein and energy content. In this transition phase three nutritional strategies are commonly used: F100, RUTF or RUTF supplemented with F75. The patient will enter the last stage of nutritional rehabilitation, the rehabilitation phase, when the child finishes all transition phase feeds and is clinically stable [3]. Mortality can reach up to 30% for complicated SAM and often occurs within the first 48 h after admission to a nutritional rehabilitation unit, i.e., during the stabilization phase [4–6]. Nevertheless, mortality is still substantial later during admission [4, 7] and even after discharge [8].

Diarrhea is prevalent among severely malnourished children and substantially increases their risk of death [4, 5, 7, 9]. Enteric infections, the underlying malnutrition and concurrent HIV disease affect the integrity, morphology and absorptive function of the intestine [10, 11]. ‘Environmental enteropathy’ can be associated with reduced dissacharidases and subsequently lead to impaired carbohydrate absorption, especially of lactose [12–15]. Importantly, reduced carbohydrate absorption can lead to severe osmotic diarrhea [16, 17].

The three main dietary regimens used in inpatient treatment of children with SAM differ substantially, especially in their carbohydrate content and composition. Lactose content is substantially higher in F100 and the overall carbohydrate intake is expected to be highest in RUTF + F75 [3]. Thus far, no studies have evaluated the different dietary approaches in the nutritional rehabilitation of malnourished children.

We performed an open-label pilot randomized clinical trial to determine whether the use of transition phase diets with different carbohydrate contents affected fecal pH, length of stay and other clinical outcomes in severely malnourished children.

Our main hypothesis was that RUTF + F75 would be associated with a higher prevalence of acidic stool.

## Methods

### Study design, outcomes and ethics statement

This open-label pilot randomized controlled trial was carried out in Blantyre, Malawi, from January to July 2013. The primary outcome of this study was fecal pH, as a measure of carbohydrate malabsorption, 3 days after the start of the transition phase. Fecal pH of ≤5.5 was defined as acidic stool and was considered suggestive of carbohydrate malabsorption [18]. Secondary outcomes included duration of stay from the first day of the transition to discharge from the ward, days with diarrhea, duration of edema, weight at discharge, hypo- and hypernatremia, reversion to F75 diet and mortality.

The sample size of 15 participants per group was based on glucose malabsorption data obtained from our previous study and calculated to detect a 20% difference in fecal pH between groups with α = 0·05 and 80% power [17]. Covariate adaptive randomization was used to achieve balance in HIV status between groups, as HIV is an important covariate. This was achieved by randomizing HIV positive and negative children separately. We performed the procedures according to the ethical standards of the Helsinki Declaration. The trial was approved by the College of Medicine Research and Ethics Committee of the University of Malawi (P.005/11/1086).

### Participants

**Fig. 1:**
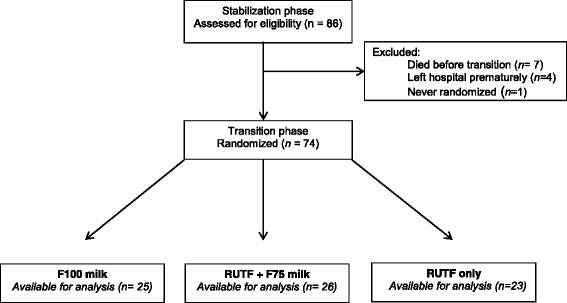
CONSORT diagram of severely malnourished children included in the study. RUTF, ready-to- use therapeutic food; SAM, severe acute malnutrition

Children were screened for eligibility at admission to MOYO house (Nutrition Rehabilitation and Research Unit of the Queen Elizabeth Central Hospital). Children aged 6–60 months, diagnosed with SAM and already admitted to the nutritional rehabilitation unit (NRU) but still in the stabilization phase were included in the TranSAM trial after written informed consent. We included both HIV positive and negative children, diagnosed by rapid antibody testing upon admission. Exclusion criteria were admission to the nutritional rehabilitation unit within the past year, severe hemodynamic instability, haematocrit level ≤ 15% and severe neurological symptoms. The study started when a child entered the transition phase. When clinically ready, a total of 74 patients were randomly assigned to one of the transition phase dietary regimens: F100 milk, RUTF only or RUTF+ F75 milk (Fig. 1).

### Randomization and enrolment

Allocation concealment was achieved by using sealed, sequentially numbered opaque envelopes containing a label for 1 of the 3 transition phase feeds. After written informed consent, when patients were ready to start a transition phase diet, guardians were asked to open the next numbered envelope showing their assigned group. The allocation sequence was computer generated by an independent collaborator. All legal guardians provided written informed consent in either vernacular Chichewa or English. The trial was registered as ISRCTN13916953.

### Study interventions

SAM patients were enrolled in the transition phase only after they had been stabilized, gained appetite and, in the case of kwashiorkor, edema had improved [3]. The full amount of the three dietary transition phase regimes were prescribed with the aim of a maximum energy intake of 135 kcal/kg/day. These regimes were based on WHO guidelines and reference tables were used to calculate the dietary amounts to be given based on body weight [3]. The first group received F100 milk at the same frequency and volume as F75 milk in the stabilization phase. The second group received RUTF given alongside with water and the amount of RUTF was increased as quickly as possible until the target intake was reached. In the third group, F75 milk was given as a top-up to RUTF to allow a gradual increase of the amount of RUTF consumed by the child as well as to ensure a minimum energy intake of 100 kcal/kg/day. We used commercially available F75, F100 and RUTF (Nutriset, Malaunay, France) (Table 1). The caregivers were given color-coded milk cards corresponding to a specific diet. Our research team was trained to distribute the correct milk formulae to participants. Intake was monitored by research nurses and assessed by the caregiver and these data were recorded daily. If a child was clinically deteriorating in the transition phase (e.g. loss of appetite; onset of one or more WHO danger signs: shock/systemic illness, respiratory distress or impaired consciousness), the patient was reverted to the stabilization phase and put back on F75 milk alone. A second transition phase was excluded from analysis, but the failure of completing the initial transition phase was documented.

**Table 1:** Nutritional composition of ‘Transition Phase’ diets^a^

	F 75	F 100	RUTF
Carbohydrates			
Lactose	11	34	19.5
Sucrose	4	9	22.9
Maltodextrin	58	9	0
Other CHO	0	0	2.1
Fat	17	33	32
Protein	6.2	12.5	13

*RUTF* ready-to-use therapeutic food, *CHO* carbohydrate

^a^ Values are represented as grams per 100 g

### Assessments

Clinical signs related to malnutrition were assessed and recorded daily by a group of 3 researchers during daily rounds, using specifically designed paper-based case report forms. The clinical signs included appetite, hydration status, degree of edema and weight. Presence of diarrhea was determined on admission to the hospital, the day before entering the transition phase (T_baseline_), the first day of transition (T_0_) and the next 3 consecutive days of treatment (T_1_-T_3_ respectively). Diarrhea was defined as 3 or more abnormal loose or watery stools. Severe diarrhea was defined as more than 10 loose or watery stools. Diarrhea was assessed by daily maternal recall. Data was entered in Access by a specific data manager and quality control was performed by another member of the research team.

Two blood samples were taken: one just before entering the transition phase and the other on transition day-3. Blood was centrifuged within 1 h of collection and plasma was stored at −80 °C for further biochemical analysis. Feces was collected on the same days and immediately stored at −80 °C.

### Laboratory analyses

Plasma sodium was measured in the clinical laboratory of University Medical Centre Groningen, The Netherlands, using standard clinical laboratory techniques. Hyponatremia was defined as a plasma sodium concentration of <130 mmol/L and hypernatremia as >145 mmol/L. We aimed to analyse reducing substances in the stool, but the only commercially available method (Clinitest, Bayer, Leverkussen, Germany) was no longer produced after the study started, therefore, we removed this outcome measure from our study. Fecal pH-measurement was performed using nitrazine paper (Micro Essential, Brooklyn, NY, USA).

### Statistical analysis

Descriptive statistics were presented as percentages for categorical variables and as mean with SD for continuous ones. Baseline differences between groups were tested using, as appropriate, Chi-square, Fisher’s exact test or two-way analysis of variance (ANOVA). The associations between transition phase diets (F100, RUTF or RUTF +F75) and the presence of acidic stool (pH ≤ 5.5), hyponatremia, diarrhea were evaluated using logistic regression. Age, sex, edema status and baseline weight-for-height Z scores were included as covariates if they contributed significantly to the models. Two-way ANOVA was used to test differences in weight at discharge and duration of stay. For analysis of hospital stay, the 6 children who died during transition were excluded. A *p* value of <0.05 was considered significant. All statistical analyses were conducted using SPSS 22.0 software (IBM, Armonk, NY, USA).

## Results

A total of 86 children were enrolled in the study, of whom 74 could be randomly assigned to a transition phase diet (Fig. 1).

Children allocated to the 3 groups were similar with respect to baseline patient and disease characteristics (Table 2). HIV status with an overall prevalence of 35% did not differ between groups. On admission, diarrhea was common and seen in 33% of patients. All randomized children remained in the study and were followed until discharge (*n* = 68) or death (*n* = 6).

**Table 2:** Patient and disease phenotype characteristics by assigned transition phase diet^a^

Characteristics	Transition phase diet			
	F 100 (*n* = 25)	F 75 + RUTF (*n* = 26)	RUTF (*n* = 23)	*P value*
Age, months	24.8 ± 10.4	23.5 ± 10.7	22.8 ± 13.9	0.84
Female, *n* (%)	14 (56)	13 (50)	13 (57)	0.74
Weight at admission, kg	7.4 ± 2.1	8.2 ± 2.5	6.9 ± 2.4	0.16
MUAC at admission, cm	11.5 ± 1.8	12.1 ± 1.8	11.1 ± 1.9	0.15
Length at admission, cm	75.7 ± 7.7	77.4 ± 9.4	73.8 ± 8.9	0.36
Weight-for-height Z score, SD	−3.2 ± 1.8	−2.5 ± 1.6	− 3.3 ± 1.7	0.21
Height-for-age Z score, SD	−3.4 ± 2.2	−2.8 ± 1.9	− 3.2 ± 2.3	0.50
Kwashiorkor, *n* (%)	16 (64)	18 (69)	15 (65)	0.92
HIV positive, *n* (%)	10 (40)	9 (35)	7 (30)	0.78
Diarrhea prior to admission, *n* (%)	16 (64)	16 (62)	17 (74)	0.63
Diarrhea day of admission, *n* (%)	6 (26)	11 (44)	6 (27)	0.30
Hyponatremia^b^, *n* (%)	4 (16)	1 (4)	0 (0)	0.27
Hypernatremia^c^, *n* (%)	2 (8)	2 (8)	2 (9)	0.27

*MUAC* mid- upper arm circumference, *RUTF* ready- to- use therapeutic food

^a^Values are n (%) or means ± SDs

^b^Hyponatremia before transition: plasma sodium concentration < 130 millimol per liter

^c^Hypernatremia before transition: plasma sodium concentration > 145 millimol per liter

### Fecal pH

**Fig. 2:**
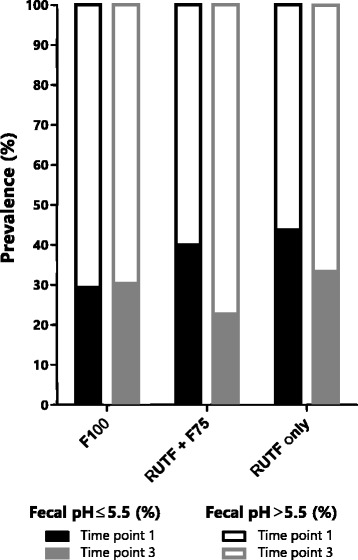
Percentage of children with acidic stool (pH ≤ 5.5) and without acid stool (pH > 5.5) for each different dietary regimen. Time point 1, day of admission, T_3_, the third day of transition

Acidic stool (pH ≤ 5.5) was present in 38% of patients on admission, and in 29% 3 days after transition, but this decrease in prevalence was not significant. Furthermore, the average fecal pH was 5.8 ± 0.7 on admission and 6.1 ± 0.7 on transition day 3. Also, stool acidity did not differ between diet groups 3 days post-transition (Fig. 2). This suggests continuing carbohydrate malabsorption during hospital admission.

### Duration of hospital stay

**Fig. 3:**
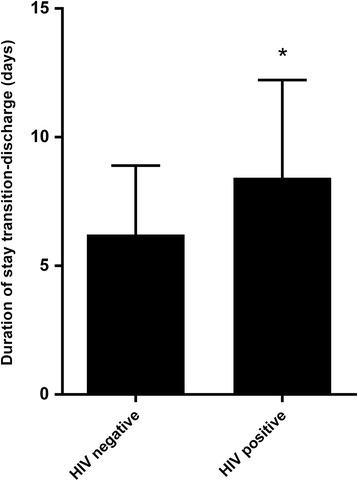
Mean duration of hospital stay for children with SAM, with and without HIV infection. Duration was measured from the first day of transition until discharge from the clinic. Values are shown as mean ± SD. Two- way ANOVA, * *p* < 0.05. RUTF, ready- to- use therapeutic food; SAM, severe acute malnutrition

On average, children were discharged 7.0 ± 3.4 days after transitioning. We did not find significant differences in duration of hospital stay between the three groups. In HIV- positive patients, the mean duration of stay from the first day of transition to higher caloric feeds until discharge was significantly higher (*P* < 0.01) compared to HIV negative patients, regardless of the intervention (Fig. 3) but this effect was only marginal after correcting for age (*P* = 0.07).

### Diarrhea

**Fig. 4:**
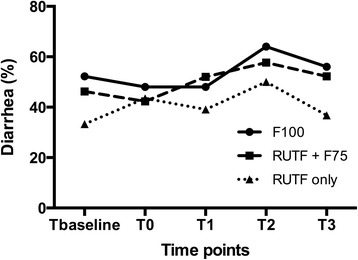
Percentage of diarrhea for different time points during the transition phase for each dietary regimen. T_baseline_, day before entering transition phase, T_0_, first day of transition phase, T_1_-T_3_ the first 3 consecutive days of treatment

Diarrhea was common in all patients irrespective of diet group but was more prevalent during the transition phase with an average of 48% compared to the first day of admission (*p* < 0.05). This study did not find differences in diarrhea prevalence between dietary regimens throughout the transition phase. The percentage of children with diarrhea did not decrease during the analysis period (Fig. 4).

### Weight at discharge and weight change

In our study, weight at discharge between groups tended to differ (P 0.09) (Table 3) but, after correcting for either initial weight, weight-for-height Z-score or edema status, this trend was not apparent. We also analysed weight gain per day in children without edema separately because loss of residual edema is expected to influence weight change in children with edema. For the non-edema group, the mean weight gain per day in the transition phase was 27.1 g in the F75 + RUTF group and 42 g in the RUTF only group, opposed to the mean weight loss of 6.6 g in the F100 group (Table 3). However, this difference was not statistically significant.

**Table 3:** Clinical outcomes by assigned transition phase diet^a^

	Transition phase diet			
	F 100 (*n* = 25)	F 75 + RUTF (*n* = 26)	RUTF (*n* = 23)	*P* value
Weight at discharge, kg	7.2 ± 2.2	7.9 ± 2.2	6.5 ± 2.1	0.09
Weight change per transition day^b^, g	−6.6 ± 63	27.1 ± 75.4	42 ± 33.5	0.34
Reverted back to F75, n%	5 (20)	1 (4)	3 (13)	0.21
Duration of edema, d	2.2 ± 2.9	2.7 ± 2.8	3.1 ± 3.6	0.61
Hyponatremia transition day 3, n (%)	5 (21)	2 (8)	2 (10)	0.35
Hypernatremia transition day 3, n (%)	1 (4)	4 (16)	5 (24)	0.16
Mortality, n (%)	1 (4)	2 (8)	3 (13)	0.52

^a^Values are n (%) or mean ± SD

^b^Weight change was only calculated for children without edema

### Reversion to F75 diet and edema

A total of 9 children had a clinical deterioration in the transition phase. Failure to transition appeared to be more prevalent in the F100 group (*n* = 5), but this was not statistically different. The mean duration of edema was similar in all groups.

### Hyponatremia, hypernatremia and mortality

Hyponatremia before transition was present in 6.9% of the patients and hypernatremia in 8.3% of patients. At the third day of transition both hyponatremia as well as hypernatremia were equally present: 12.9%. In this study, there were no statistically significant differences between the groups. Mortality was 8% (*n* = 6) during the transition phase to discharge. The mortality did not significantly differ between the three treatment groups (Table 3).

## Discussion

To our knowledge, this study is the first to do a head-to-head comparison of the three most commonly used dietary regimens after initial stabilization of severely malnourished children. The results from our pilot study do not support the hypothesis that nutritional rehabilitation strategy with the highest carbohydrate content is associated with a higher percentage of patients with an acidic stool.

Studies have shown that diarrhea is very prevalent in severely malnourished children and is associated with worse clinical outcome [4, 5, 7, 9]. In low-resourced countries diarrhea is often thought to be associated with intestinal infections [7]. In addition, malnutrition itself has also been associated with impaired integrity and absorptive function of the intestine [10, 11]. Studies have indicated that carbohydrate malabsorption in general and lactose intolerance specifically is prevalent in children with SAM [13, 15, 19]. Based on small-bowel biopsies, reduced levels of dissacharidases, such as lactase, have been observed in children with SAM [12, 19]. In addition, monosacharide malabsorption has also been found in children with SAM [17, 20]. Malabsorption of these carbohydrates is well known to induce osmotic diarrhea [16, 17, 21], but only a few studies have focused on the possible relation between diet composition and diarrhea in children with SAM [15, 19, 22].

Based on these studies we expected that providing severely malnourished children with a combination of RUTF + F75 (with potentially the highest carbohydrate content, depending on intake of RUTF) or F100 (highest lactose content) would lead to a higher incidence of acidic stool (pH ≤ 5.5), diarrhea and a prolonged hospital stay. Acidic stool (pH ≤ 5.5), indicative for carbohydrate malabsorption, was present in 38% of the children in our study on admission and was 29% after 3 days of transition. This is similar to the 26% carbohydrate malabsorption in malnourished children previously reported [23]. Although we were unable to detect differences between groups, the mean fecal pH was nevertheless greater than 5.5 in our study, which supports results obtained from other studies of malnourished children [11, 22]. This may be explained by a number of things. First, this was a pilot study with a relatively small sample size. In our study 33% of severely malnourished children presented with diarrhea, which is lower than the 50–67% described in previous literature [5, 9]. Although still substantial, this relatively lower prevalence of diarrhea in our cohort could have influenced the power to detect differences in this outcome. All three nutrition rehabilitation diets contain a high amount of carbohydrates and the differences in composition may not vary enough to induce changes in the presence and severity of diarrhea or in the overall clinical outcome.

We did not find a significant effect of different transition phase diets on hospital stay. However, the presence of HIV infection may increase the duration of hospital stay. HIV infected malnourished children have a varied clinical presentation [6] and the underlying pathophysiological mechanisms are potentially different from children without HIV, although this is hardly studied [24]. Superimposed infections, including diarrhea, tuberculosis and extensive cutaneous infections are common [25, 26] and complicate not only the clinical picture but also the therapeutic response of children with SAM [27].

Disturbances in electrolytes are common in children suffering from severe acute malnutrition. Electrolyte disturbances can occur as a result of diarrhea, vomiting and/or dehydration [28]. Clinically relevant hyponatremia was present in 6.8% of the admitted children with SAM in our study. The prevalence of hypernatremia at admission was 10,8%, which was lower than the previously reported 15.7% with the same cut- off value [28]. Before transitioning (T_0_), hyponatremia was present in 6.9% of the patients and hypernatremia in 8.3% of the patients. Interestingly, 3 days after transition (T_3_) the percentage of patients with hyponatremia and hypernatremia was 12.9% in both groups. This coincided with an increase in diarrhea. The lack of improvement in these clinical and biochemical parameters over time suggests an on-going enteropathy, perhaps in part related to persistence of intestinal infections. This could also be related to diet-induced osmotic diarrhea due to general malnutrition-related impairment in intestinal function. Finally, all children receive antibiotic treatment which could be involved in the on-going diarrhea. Total mortality after initial stabilization was still substantial in our study (8%). This is in line with previously reported case series [4, 29, 30]. We did not find difference in mortality between our interventions but our study was not powered to detect differences in mortality rates.

This study shows that performing a trial to evaluate these different dietary interventions is feasible. However, there are a number of limitations that would need to be addressed. The sample size needs to be adequate to detect differences in important outcomes such as mortality. Secondly, challenges such as monitoring stool frequency would need to be mitigated. Providing 24-h direct monitoring of stool frequency would be ideal as maternal recall, although commonly used, can be at times unreliable and is prone to recall bias. Furthermore, we recommend monitoring and supporting the caregivers during feeds, this would avoid cases were caregivers may be switching or mixing diets with those of other patients. This trial was an *effectiveness* trial performed in a “real world” low income setting with limited availability of research support staff. Also, weight changes in the transition phase can be influenced by hydration status and changes in edema for children with kwashiorkor who often have not lost all their edema at that time. Fully documenting these variables would be useful. In the trial design ‘recovery rate expressed in incremental weight/kg body weight/day was also listed as outcome measure. However, this recovery rate was often unreliable as children were discharged without increase in body weight (especially in kwashiorkor). Without stringent budget restrictions, it would have been interesting to evaluate the effect of the three diets on microbiota as this is increasingly recognized to play a role in SAM.

## Conclusions

In conclusion, the results from this clinical trial do sufficiently support the hypothesis that the varying nutrient composition of three commonly used dietary rehabilitation regimens (F75, F100 or RUTF) lead to differences in outcomes in children with SAM. The abnormal stool frequency and composition observed throughout hospitalization suggest a sustained enteropathy. Future clinical trials focused on diarrhea should aim to include more objective evaluation of stool frequency and consistency to not rely on maternal recall and include objective outcome measures of intestinal function, such as macronutrient loss using bomb calorimetry or carbohydrate content in stool using, for example, mass spectrometry.

## Abbreviations

F100: Formula-100, therapeutic milk
F75: Formula-75, therapeutic milk
RUTF: Ready-to-use therapeutic food
SAM: Severe acute malnutrition
